# Infrared Studies in the 1- to 15-Micron Region to 30,000 Atmospheres

**DOI:** 10.6028/jres.063A.003

**Published:** 1959-08-01

**Authors:** C. E. Weir, E. R. Lippincott, A. Van Valkenburg, E. N. Bunting

## Abstract

A pressure cell was constructed using a pair of type II diamonds for study of infrared spectra of solids in the 1- to 15-micron region. Using commercial infrared equipment, spectra can be studied routinely to calculated pressures as high as 30,000 atmospheres. Under pressure, bands generally shift to higher frequencies and decrease in intensity. The magnitude of both changes depends on the mode of vibration. Occasionally major changes in spectra occur. In calcite the carbon-oxygen symmetric stretching, mode *v*_1_, becomes active at elevated pressures while the doubly degenerate *v*_3_, stretching, and *v*_4_, bending, frequencies split. From the shift in frequency of *v*_1_ with pressure the “compressibility”, [(−1/R_o_) (dR/dp)], of the C—O bond length, *R*, is calculated to be 2.8×10^−7^/atmosphere. Major spectral changes are not observed in the same pressure ranges in other carbonates having the calcite or aragonite structures. The results for calcite may be explained by a shift of the ${\text{CO}}_{3}^{=}$ ion from the trigonal axis under pressure.

## 1. Introduction

In recent years the short range interatomic forces and perturbing effects of neighboring atoms on each other have become of increasing interest. The perturbation is a function of the interatomic distance and any serious study of this effect in condensed systems requires measurements involving systematic changes in the spacings. Two parameters are immediately available for systematically varying the interatomic distances in a given structure—pressure and temperature. The variation produced by temperature changes is limited by the expansivity of the material and for solids cannot exceed the limits imposed by the melting point and the absolute zero of temperature. For some purposes wide changes in temperature are undesirable because of the concomitant change in the thermal (kT) energy involved. Limited studies of such nature have been made [1].^2^ The effect produced by changes in pressure is of considerably more interest since relatively large changes in spacing may be produced with moderate pressures, i.e., 50,000 atm, with no accompanying change in the kT energy. Drickamer and his coworkers [2,3,4] have studied such effects by the infrared absorption method to pressures of 12,000 atm, but with a 0.2*μ* to 4*μ* spectral range imposed by the cutoff of the sapphire windows used in their apparatus. The frequencies for many important modes of vibration are not found in this spectral region and the present investigation was undertaken to extend the region studied farther into the infrared and if possible to higher pressures.

## 2. Apparatus

### 2.1. Pressure Cells

For infrared studies at high pressures the window is of prime importance. For transmission purposes the alkali halides are ideal but they are mechanically weak and not generally suited for pressure work. It is noted that Drickamer [5,6] has evolved an ingenious bomb design, using an alkali halide simultaneously as the pressure medium and the window, which is useful to pressures as high as 200,000 atm, but so far he has restricted his studies to the higher frequencies. An alternative material is available which combines high strength with excellent transmission, i.e., diamond.

**Figure 1:**
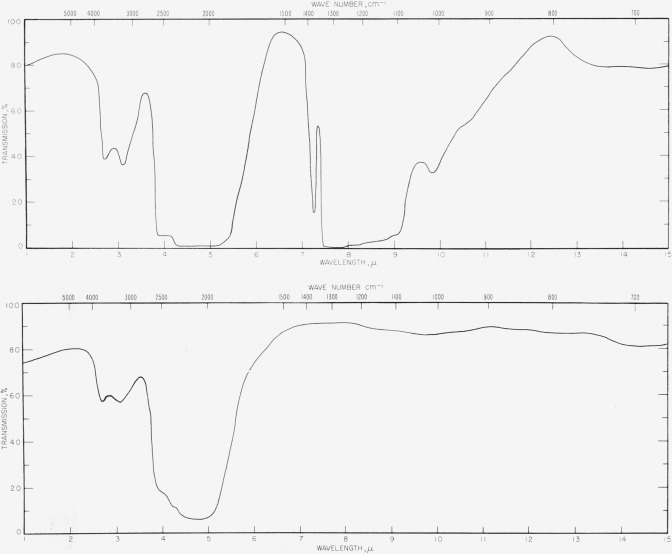
Infrared transmission spectra for typical type I (*above*) and type II (*below*) diamonds.

Natural diamonds may be classified into two main categories known as types I and II. Type I diamonds comprise at least 98 percent of all diamonds and are not particularly useful for windows since they contain strong absorption bands in the infrared. Type II diamonds, however, are relatively transparent with the exception of a strong absorption band near 5*μ*. In practice many subclassifications of both types are recognized [7] which show differences in absorption, but very transparent type II diamonds are available which may be used between 1*μ* to 4*μ* and 5.5*μ* to 15*μ* as infrared windows. The explanation for the differences between the absorption properties of the diamonds is not known [7]. Figure 1 shows typical transmission curves for a type I diamond and for a type II diamond used as a pressure cell. In connection with figure 1, the path length in the diamond is of the order of ¼ in. Thinner diamonds might be quite useful even near the region of the 5*μ*-absorption band.

**Figure 2:**
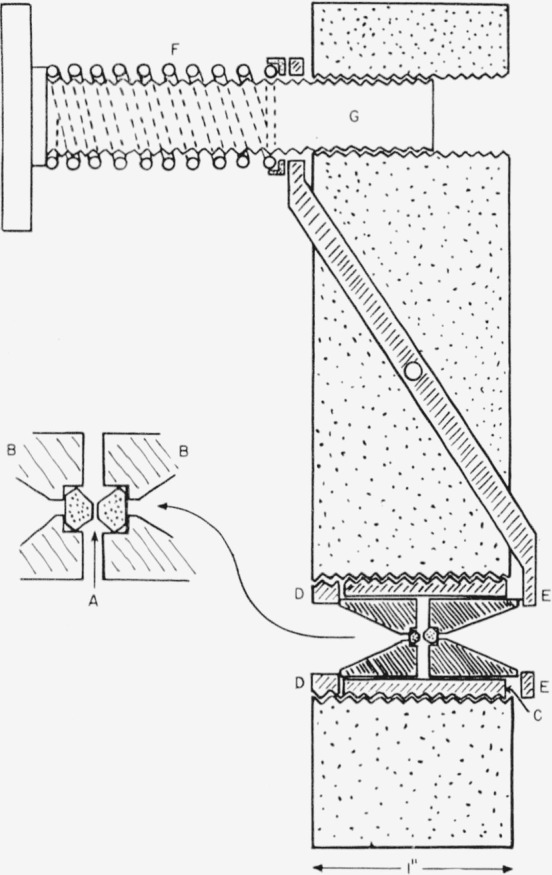
Schematic diagram of diamond “squeezer” for infrared transmission studies to 30,000 atm.

In order to attain maximum pressures with simple equipment the “squeezer” design developed by Bridgman [8] was used. In this device a uniaxial force is applied to a specimen contained between two flat surfaces. The apparatus used is shown in figure 2. Two gem-cut type II diamonds, each weighing 0.036 g, comprise the squeezer anvils. The culets of each diamond were ground off to form small flats parallel to the tables. The specimen is placed between these small flats which have an area of approximately 0.0002 in.^2^ Each diamond, A, is seated on its tabular face which rests in a close-fitting recess in a stainless-steel piston, B. Each piston is drilled longitudinally with a hole 0.060 to 0.075 in. and is bored out with a tapering hole which extends to within 116 in. of the diamond. This taper is designed to permit acceptance of the maximum flux from a convergent cone of radiation which passes through both pistons, the diamonds, and the specimen contained between the diamond surfaces. The specimen itself is located at the focus of this beam.

The pistons are free to slide in a dural bearing, C, that screws into a large block of steel carrying the pressure generating equipment. The threaded mount is used to permit ready interchange of bearings when desired. One piston rests against a thrust bearing, D, which also screws into the steel block. At the other end a presser plate, E, bears against the other piston. The presser plate is connected to a lever that is pivoted in the block, and actuated by a calibrated spring, F, which bears against the upper end of the lever. The presser plate is bored out to permit entrance of the convergent cone into the piston. In operation the position of the pistons may be varied by moving the thrust plate so that the presser plate is perpendicular to the axis through the diamonds. This positioning ensures the absence of components of force at right angles to the thrust axis. The spring is compressed by means of a manually operated screw, G, having 20 threads/in. In the present device the lever arms are of equal length, but inasmuch as there is negligible motion of the pistons, the lever arms may be varied to produce different pressure ranges using the same spring. The whole unit is designed to be mounted in a commercial infrared-beam condensing unit, and the cell is only 1 in. in length to fit into the highly restricted focal area of the lens system.

The load is determined by measuring the compression of the spring. The pressures are calculated values obtained by dividing the load by the area of the smaller of the two diamond faces, neglecting frictional forces. No greater precision is required at the present time. Experiments have shown that the thrust transmitted by the specimen may be measured by determining the resistance of a small coil of mangan in wire placed under thrust plate D, if greater precision is desired.

The diamond-bearing surfaces are irregular octagons. The lengths of each side and the external angles were measured using a micrometer eyepiece on a rotating stage microscope. The data were laid out on cross section paper and the areas of the bearing surfaces determined by counting the squares. Two cells have been used with the smaller diamonds having measured areas of 0.000156 in.^2^ and 0.000182 in.^2^

### 2.2. Diamond Grinding

The diamonds were ground using a porous cast-iron lap charged with 5*μ* diamond dust. A 0.5-in.-diam shaft was mounted parallel to but eccentric to the axis of the lap. The diamonds were mounted on the face of bronze-bearing stock which was machined to permit free rotation on this shaft with no apparent wobble. Diamonds were ground in pairs and were soldered to the smooth face of the bearing stock in diammetrically-opposed positions. In mounting, the diamonds were seated on their tabular faces and weighted down with a piece of metal placed on the culet. A close-fitting, sleeve on the outside of the bearing together with a plug in the bore of the bearing served to produce a cup surrounding the diamonds. The bearing was heated and the cup filled with soft solder to firmly embed the diamonds. Removal of the sleeve and plug and filing off the excess solder completed the preparation for grinding. The grinding operation required about 2 hr and produced a highly polished flat surface parallel to the tabular seat. During grinding the diamonds rotated against the lap, and the bearing was loaded with additional weight as soon as the sharp culets were ground off. To utilize the massive support principle and to minimize alinement problems, the two opposing diamond faces were purposely made of different areas.

### 2.3. Infrared Equipment

A commercial double-beam infrared spectrometer equipped with a beam-condensing unit was used to cover the range 1*μ* to 16*μ*. The focussing device produced an image of the slit 1.5-mm wide and 1-cm long. Since the aperture of the hole at the base of the diamond was approximately 2mm in diameter, essentially the full width of the beam was used but only a small portion of its length was accepted by the cell. The loss in available energy necessitated restriction of the reference beam to permit utilization of the full scale of the instrument. This adjustment was accomplished with a sheet of aluminum perforated with a number of holes, the number of holes being adjusted to give 100-percent transmission in regions of minimum absorption. The small amount of energy available necessitated slow scanning. In all experiments the scanning rate was fixed at 0.5*μ*/min.

## 3. Experimental Method

The substance to be studied was generally ground to a fine powder. The piston with the smaller diamond (the entrance pupil) was inserted in the bearing and a small quantity of powder was placed on the surface with a small spatula. The other piston was inserted and the thrust plate screwed in place. The pressure was raised to a few thousand atmospheres to produce a clear film between the diamond faces. The unit was then placed in the focal point of the lens system and its position adjusted in the beam to produce a maximum transmission in a spectral region containing no strong bands. The pressure was then reduced to a low value (ca. 3,000 atm) and the spectrum scanned completely. The pressure was raised to a higher value and the process repeated until the maximum pressure was reached. An arbitrary maximum calculated pressure of 31,000 atm was set in these experiments because preliminary studies indicated possible incipient failure of the diamonds at about this pressure. The pressure cell can be set up on the microscope stage, and the diamonds and the specimen may be observed at all pressures at low magnifications. Slight modification of the cell or use of long focal-length objectives would permit higher magnification. Frequent microscopic examinations were made to examine the diamonds and the uniformity of the film of material on the diamond surfaces.

To study strong absorption bands in detail some materials were diluted with KBr or LiF. Dilutions were generally made by grinding the components either in 1 to 1 or 2 to 1 proportions and proceeding as before. It was found that KBr extruded rapidly under pressure and was not satisfactory. Lithium fluoride was a very satisfactory medium with little or no extrusion, but in dilute mixtures evidence of interaction with the dispersed substance has been observed in some instances. With LiF the complete range may not be studied as this material absorbs strongly above 14.5 *μ*.

### 3.1. Pressure

Some consideration of the hydrostaticity of the pressure on the specimen is necessary but only a qualitative discussion is possible at this time. In similar squeezers using Carboloy, the anvils become concave on continued use and eventually confine the specimen in an enclosed capsule. Under these circumstances one may be tempted to consider the specimen as subjected to reasonably hydrostatic pressure. Even under these circumstances, however, there is considerable question as to the hydrostaticity of the pressure [9,10]. In the diamond squeezer no plastic deformation of the surfaces has been observed, and there is considerably more uncertainty of the hydrostaticity of the stress on the specimen. Marked lateral-extrusion tendencies, observed as changes in intensities, have been noted in some specimens. With diluents the pressure on the specimen is more apt to be considered hydrostatic, but completely concordant data are obtained with or without diluents. On the other hand, no transition reported under hydrostatic pressure has been identified.^3^ However, transitions involving small displacements of ions are not expected to produce major changes in the infrared pattern in the frequency range studied here. The frequencies of internal modes observed in this region will not be primarily affected and the wavelength is too long to expect much scattering loss from interfaces of small crystals. All effects observed appear to be reasonably reversible.

Therefore, although the uncertainties are fully recognized, the stress applied to the specimen will be considered to be a pressure and no further discussion of its hydrostaticity will be made here.

## 4. Discussion of Some Initial Results

The infrared spectrum of a given substance may show a number of changes on application of pressure. These include red or blue shifts of frequencies from their positions at 1 atm, the occurrence of new bands, the splitting of degenerate bands arising either from a change in selection rules or from an enhanced interaction of the lattice modes with the internal molecular modes, and changes in apparent absorbance resulting either from a broadening of the band under pressure or from a change in absorptivity of the band. Numerous examples of the effect of pressure on the positions of infrared bands below 5 *μ* have been given by Drickamer and his coworkers [2,3,4]. We will illustrate a number of the changes with pressure in the spectra of CaCO_3_ (calcite and aragonite).

### 4.1. Calcite

Calcite, CaCO_3_, has been the subject of a number of infrared studies [1,11,12,13,14,15,16]. Today its vibrational spectrum is considered to be rather well understood both in terms of frequency assignment and in the effects of the crystal symmetry [1,11,12,13,14]. The main features of the spectrum are summarized and will be discussed in terms of the correlation diagram shown in table 1 [1].

The free carbonate ion, ${\text{CO}}_{3}^{=}$, has D_3_*_h_* space symmetry with four internal frequencies corresponding to a symmetric stretching, *v*_1_; an out of plane bending, *v*_2_; a doubly degenerate stretching, *v*_3_; and a doubly degenerate bending, *v*_4_. For D_3_*_h_* symmetry the infrared active species are $A_{2}^{\prime \prime }$ and E′, while for the site symmetry D_3_, the infrared active species are A_2_ and E. Thus, three of the four internal frequencies of ${\text{CO}}_{3}^{=}$ should be observed while the symmetric stretching frequency, *v*_1_, should remain inactive. However, degenerate lattice frequencies of translational or rotational origin may interact with the degenerate fundamental internal frequencies to produce a splitting or doubling [12, 13]. It has been reported that splitting has been observed in single crystals of calcite [12], but it is usually not found in pellet or mull spectra of calcite [17,18].

**Figures 3:**
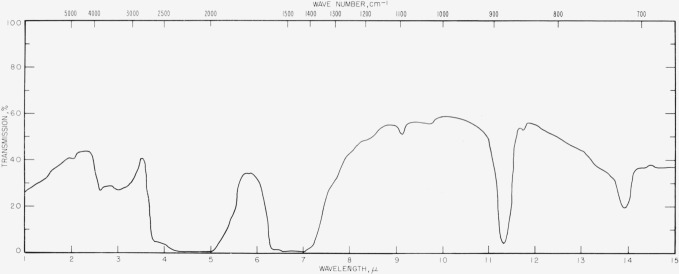


**Figures 4:**
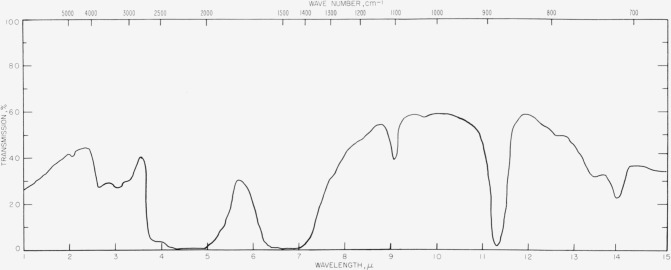


**Figures 5:**
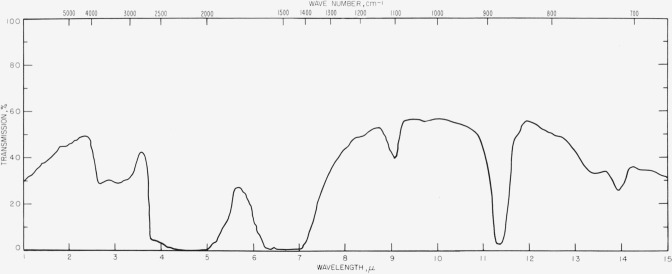
Infrared transmission spectra for calcite at various pressures. Top, 3,000 atm; center, 18,000 atm; bottom, 31,000 atm.

**Figure 6:**
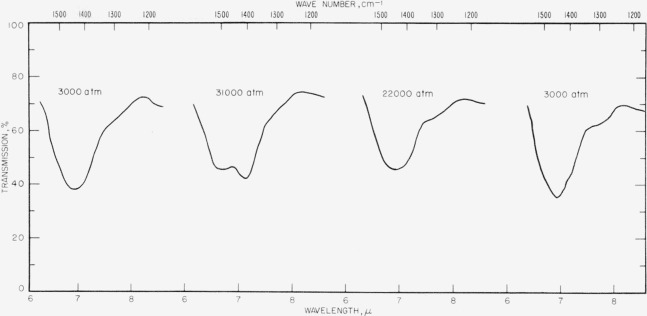
Detailed study of 1,463 cm^−1^ band of calcite using *LiF* diluent.

The spectra of calcite at various pressures are shown in figures 3, 4, 5, and 6. One effect of pressure on the vibrational spectrum is observed to be an enhancement of the splitting of the degenerate fundamentals. In figure 6 the *v*_3_ fundamental at 1,463 cm^−1^ at 3,000 atm is split into two components under a pressure of 31,000 atm which are separated by 103 cm^−1^. This splitting is reversible and disappears when the pressure is lowered. The splitting of *v*_4_. is less apparent but we have tentatively assigned the new band appearing at 748 cm^−1^ in figures 4 and 5 at high pressure as one component of the split *v*_4_ band. This process is also reversible and the 748 cm^−1^ band disappears as the pressure is lowered. The *v*_2_ mode at 883 cm^−1^ is not degenerate and shows no tendency toward splitting.

Evidence that the ${\text{CO}}_{3}^{=}$ ion sites may be deviating from D_3_ symmetry is furnished by the appearance at 1097 cm^−1^ of the forbidden symmetric stretching frequency, *v*_1_, at elevated pressures. This band shows a marked increase in intensity as the pressure rises, which is interpreted as a gradual relaxation of selection rules arising from either a change in symmetry or an increased perturbation of the internal frequencies by the more intense crystal field. The increased intensity of *v*_1_ could be due to a change of phase but we have been unable to detect evidence in the infrared spectrum of a definite phase change from the characteristics of the spectrum.

**Figure 7:**
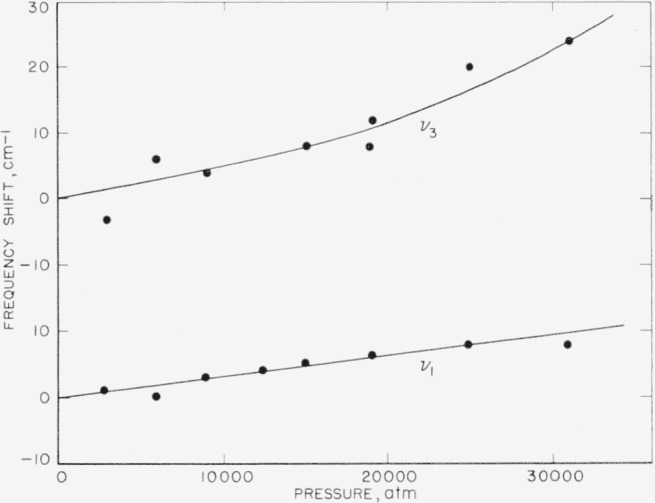
Shift of center of gravity of v_1_ and v_3_ bands of calcite with pressure.

With increasing pressure there is a definite blue shift of *v*_1_ and a similar blue shift of the center of gravity of the two components of the degenerate stretching frequency *v*_3_. The out-of-plane bending frequency *v*_2_ does not shift significantly. The shifts of *v*_1_ and *v*_3_ are plotted as a function of pressure in figure 7.

The shift of the stretching frequencies can be interpreted as the result of a decrease in the C—O bond distance in the ${\text{CO}}_{3}^{=}$ ion under pressure. To calculate the change of bond length, use is made of the internuclear potential function [19, 20].

$$V=D_{e}\;\left[1-e^{-n\Delta R^{2}/2R}\right],$$ (1)

where

*D_e_*=bond dissociation energy,
*R_e_*=equilibrium bond length,
Δ*R=R−R_e_*=change in bond length from the equilibrium value,
*n*=*K_e_R_e_/D_e_*, and
*K_e_*=bond stretching force constant.

This potential function has been found useful in predicting and correlating bond properties in both polyatomic and diatomic molecules. The change in bond lengths may be calculated by using eq (1) to calculate *dV/dR* and *d*^2^*V/dR*^2^ followed by substitution of the known C—O distance and the observed *v*_1_ frequency shift.

$$dV/d\;R={\frac{D_{e}ne}{2}}^{-n\Delta R^{2}/2R}\;\left[\frac{2\Delta R}{R}-\frac{\Delta R^{2}}{R^{2}}\right],$$ (2)

and, neglecting terms in Δ*R*^2^ and higher orders of Δ*R*,

$$k=d^{2}V/d\;R^{2}\approx \frac{D_{e}n}{2}\;\left[\frac{2}{R}-\frac{4\Delta R}{R^{2}}\right],$$ (3)

$$\frac{k-k_{e}}{k}\approx \frac{-2\Delta R}{R^{2}}\;R_{e}\approx \frac{\Delta k}{k}=-\frac{2\Delta R}{R},$$ (4)

and since *R_e_* ≈ *R*

$$\frac{\Delta k}{k}=\frac{2\Delta v}{v}$$ (5)

or

$$\frac{\Delta R}{R}=-\frac{\Delta v}{v}.$$ (6)

**Figure 8:**
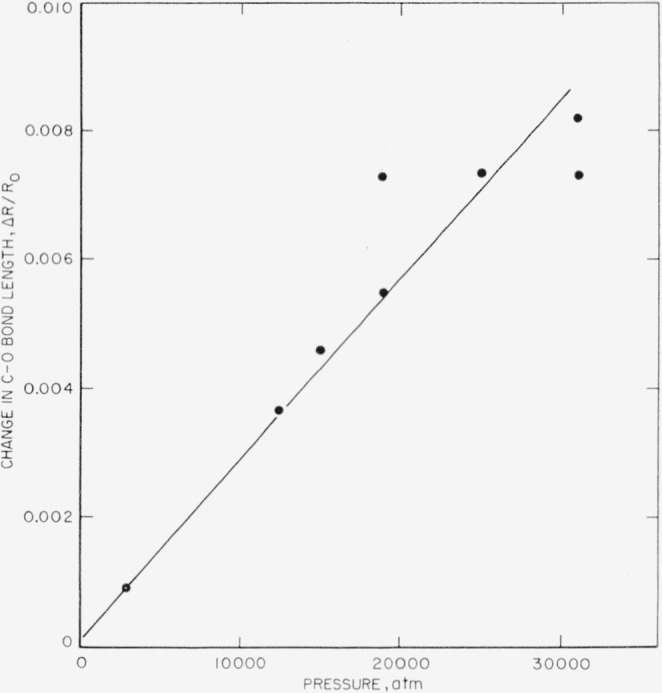
Change in *C*—*O* bond length of calcite with pressure.

The calculated change in bond length is plotted against pressure in figure 8. From figure 8 it will be noted that the C—O bond is rather incompressible since −Δ*R/R* is quite small. However, the value of [(−1/*R*)(Δ*R*/Δ*P*)] calculated, assuming linearity at low pressures, is found to be 2.8×10^−7^/atm. This figure is of quite reasonable order of magnitude and essentially the same as the compressibility of calcite perpendicular to the trigonal axis reported by Bridgman [21].

**Figure 9:**
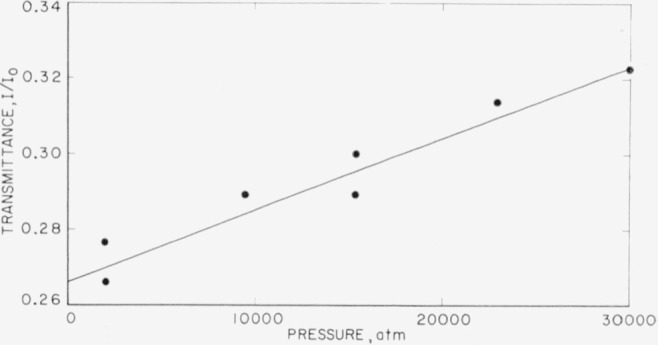
Transmittance of v_3_ band of calcite at different pressures.

**Figure 10:**
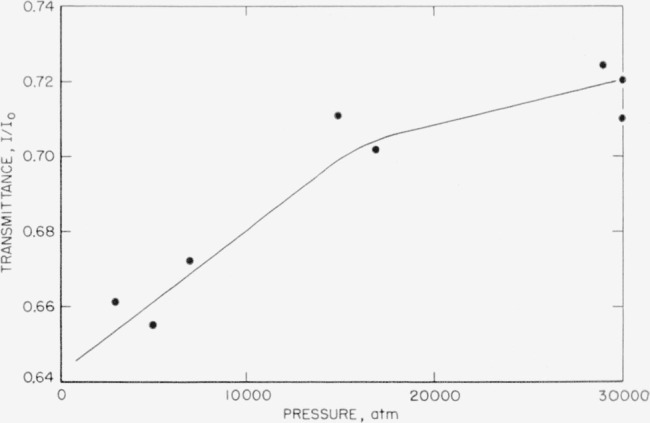
Transmittance of v_4_ band of calcite at different pressures.

Another effect of pressure on the fundamental spectrum of calcite is a noticeable change in apparent absorbance, with most bands appearing weaker at high pressures. This effect appears quite general and has been observed on most substances studied to date. The exceptions usually are cases where forbidden frequencies are appearing because of a change of selection rules arising from the effect of pressure on the crystal field as in the case of the symmetric stretching frequency of the ${\text{CO}}_{3}^{=}$ ion. In order to measure intensity changes quantitatively, considerable care must be exercised because of the tendency of specimens to extrude under pressure. A consistent set of measurements involves subjecting specimens to several pressure cycles to obtain concordant values of *I*/*I*_0_ which are free from errors due to extrusion. It has been found that the best results are obtained by systematic studies of a single band rather than of the complete spectrum. In figures 9 and 10 are plotted such measurements of the transmittance, *I/I*_0_, against pressure for the *v*_3_ and *v*_4_ bands of calcite. The change with pressure may be due in part to a broadening of the band with increasing pressure as well as to a change in absolute intensity of the band. The data are not sufficiently precise to show much more than qualitative behavior. The discontinuity of slope shown in figure 10 is real and is associated with splitting of the *v*_4_ band. There are insufficient data to define clearly the shape of the curve, but the trend to a decreased intensity with increased pressure is apparent.

### 4.2. Other Carbonates

Aragonite has a crystal symmetry corresponding to the space group $V_{h}^{16}$ with four molecules per unit cell. Calcium ions are located on sites of symmetry *C_i_* and carbonate ions on sites of symmetry *C_s_* [13]. Six internal frequencies of ${\text{CO}}_{3}^{=}$ are now permitted in the infrared, two additional frequencies being derived from the *v*_3_ stretching and *v*_4_ bending modes respectively through the removal of degeneracy and one from the *v*_1_ mode that is now active. Potassium bromide pellet spectra of aragonite at 1 atm show that *v*_4_ is split by 13 cm^−1^ but no detectable splitting of *v*_3_ is observed. Failure to observe the splitting of *v*_3_ may be due to the broadness and high intensity of this band.

Spectral data at elevated pressures have also been obtained on carbonates having both the calcite and aragonite structures. In none of these is an additional resolved splitting noted at elevated pressures over that observed at 1 atm, although with magnesite (MgCO_3_ which has the calcite structure) there is some indication that splitting of the *v*_3_ band may be occurring at the maximum pressures. The *v*_1_ band does not appear in magnesite, however. In aragonite the *v*_1_ band is observed as predicted by the selection rules but it does appear to change appreciably in intensity as the pressure increases. There is also no observed splitting of *v*_3_ under pressure. The shifts of bands with pressure are of approximately the same magnitude as with calcite.

A complete explanation of the observations must await studies at somewhat higher pressures. It would appear that if the ${\text{CO}}_{3}^{=}$ of calcite were displaced from the trigonal axis under pressure, the *v*_1_ band would appear and both *v*_3_ and *v*_4_ would split. A similar effect might not occur at comparable pressures in magnesite since it is expected to be less compressible. We have no ready explanation for the fact that the *v*_3_ band of aragonite shows no evidence of splitting.

Additional studies have been made on many other compounds both organic and inorganic with results generally similar to those reported here. These data will be compiled and reported in the future.

## Figures and Tables

**Table 1:** a Infrared correlation diagram for calcite

Internal frequency	Lattice b frequency	Molecular symmetry and species	Site symmetry and species	Factor symmetry and species
		
		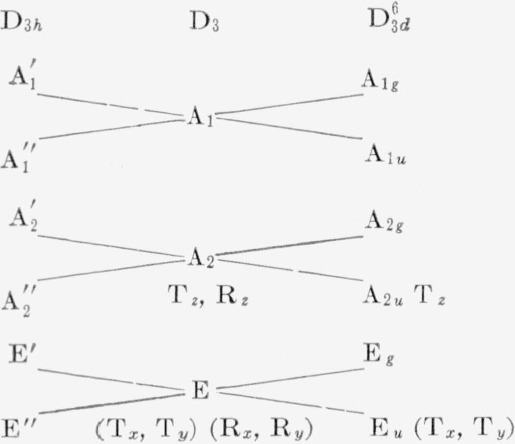		
*v*_1_				
	R*_z_*			
	T*_z_*			
*v*_2_	T*_x_*, T*_y_*			
*v*_3_, *v*_4_	R*_x_*, R*_y_*			

${\text{CO}}_{3}^{=}$ Internal frequencies

*v*_1_—1,087 cm^−1^—symmetric stretching

*v*_2_— 879 cm^−1^—out-of-plane bending

*v*_3_—1,432 cm^−1^—asymmetric stretching, doubly degenerate

*v*_4_—714 cm^−1^—planar bending, doubly degenerate

a See reference [1].

b R and T represent lattice frequencies of rotational or translational origin.

## Footnotes

2 Figures in brackets indicate the literature references at the end of this paper.

3 Possible transitions have subsequently been observed in NaNO_2_ and C_6_H_5_ COOH.
